# Rediscovery and amended descriptions of *Begonia kingdon-wardii* (Begoniaceae) from North Myanmar

**DOI:** 10.3897/phytokeys.94.21753

**Published:** 2018-01-29

**Authors:** Wen-Hong Chen, Xiao-Hua Jin, Yu-Min Shui

**Affiliations:** 1 Key Laboratory for Plant Diversity and Biogeography of East Asia, Kunming Institute of Botany, Chinese Academy of Sciences, Kunming 650201, Yunnan, China; 2 Institute of Botany, Chinese Academy of Sciences, Beijing 100093, China; 3 Southeast Asia Biodiversity Research Institute, Chinese Academy of Sciences, Yezin, Nay Pyi Taw 05282, Myanmar

**Keywords:** *Begonia*, *Begonia kingdon-wardii*, *Begonia* sect. *Sphenanthera*, Myanmar, Rediscovery

## Abstract

*Begonia kingdon-wardii* Tebbitt was rediscovered in 2014 from Myanmar after 67 years based on its last collection in 1937. Its previously unknown female flower and inaccurate morphology of leaf and ovary have been additionally described. This species belongs to Begonia sect. Sphenanthera (Hassk.) Warb. due to its dioecious habit, 3-locular ovary, berry fruits and thick placenta segments. Morphologically, it is similar to *Begonia gulinqingensis* S. H. Huang & Y. M. Shui in the leaf shape, placentation and fruit shape, but different in its dioecious plants, pliciform leaves, two-petalled female flowers and berry fruits. The rediscovery of this amazing living species will attract significant interest for scientific research and horticultural application.

## Introduction

*Begonia* L. includes more than 1800 species which are widely distributed in tropical and subtropical areas (Ku et al. 2007; Hughes et al. 2015). During a recent botanic survey on plant diversity of Hkakaborazi National Park in North Myanmar in 2014, a dioecious species was collected of *Begonia* with 3-loculed berry fruits and was unknown by the comparison with the report of the floristic report in this region (Khin and Aung 2002). According to the treatment of sections in *Begonia* (Doorenbos et al. 1999; Shui et al. 2002), this species should belong to Begonia sect. Sphenanthera (Hassk.) Warb. Based on further comparison with all the previously published species in Begonia sect. Sphenanthera (Smith et al. 1986; Ku et al. 1997; Pham1999; Huang and Shui 2006; Hughes 2008; Averyanov and Nguyen 2012), its two-petalled female flower and the serpentinous adaxial leaf surface are distinct and unique in this section.

**Figure 1.**
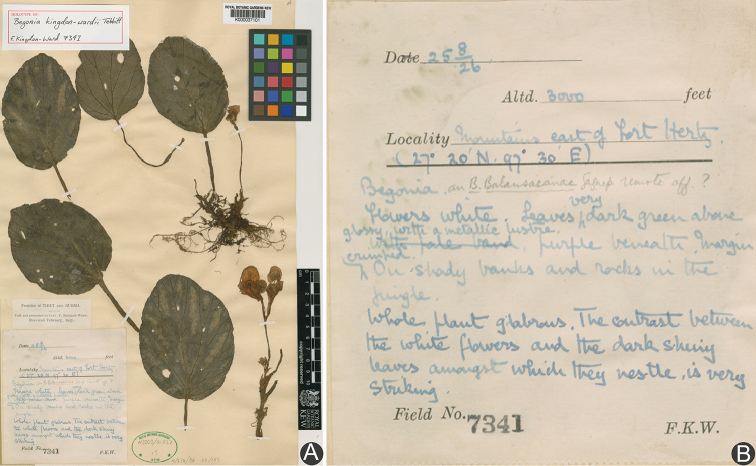
Holotype of *Begonia kingdon-wardii* Tebbitt (**A**) and the annotation on the holotype specimens (**B**).

*Begonia kingdon-wardii* was first reported in 2007 based on the holotype specimen collected from Northern Myanmar in 1926 (Fig. 1) and a paratype specimen collected near its type locality in 1937. Thereafter, no more specimens of this species had been found until 2014 subsequent to examination of the main herbaria worldwide which possessed rich collections from the regions, such as E, K, NY and so on (Hughes et al. 2015). Therefore, the species was rediscovered 67 years later in the field after the last specimens had been collected in 1937 (Tebbitt 2007). In the protologue, there were no detailed descriptions of petals on the female flower and a wrong description with 4-locular ovary, which is actually 3-locular ovary according to the authors’ observation in the field.

*Begonia kingdon-wardii* was named after Frank Kingdon-Ward, a famous plant hunter in the earlier period of the 20^th^ century. For the purpose of collecting seeds of beautiful hardy plants and of dried specimens for English gardens, he carried out many expeditions in N Myanmar, NE India (Assam) and SW China (SE Tibet and NW Yunnan) from 1911 (Lyte 1989, Tebbitt 2007). Furthermore, he wrote and published 25 books, such as “On the Road to Tibet" (1910), “In Furthest Burma” (1921), “Riddle of the Tsangpo Gorges” (1926), “Plant Hunting on the Edge of the World” (1930), “Burma’s Icy Mountains” (1949) etc. Up to now, more than 100 species have been named after him, such as *Aralia kingdon-wardii* J. Wen, Lowry & Esser, *Cinnamomum kingdon-wardii* Kosterm., *Daphne kingdon-wardii* Halda., *Euphrasia kingdon-wardii* Pugsley, *Lilium wardii* Stapf ex W. W. Sm., *Impatiens kingdon-wardii* Nob. Tanaka & T. Sugaw., *Ixora kingdon-wardii* Bremek., *Mussaenda kingdon-wardii* Joyaweera, *Rhododendron wardii* W. W. Sm., and *Vaccinium kingdon-wardii* Sleumer.

### Begonia kingdon-wardii

Taxon classificationPlantaeCucurbitalesBegoniaceae

Tebbitt in Kew Bulletin 62: 143, 2007

Figs 1A , 2

#### Type.

Myanmar, Kachin Mts E of Fort Hertz, 27°20'N, 97°30'E, alt. 900 m, Aug. 1926, Kingdon-Ward 7341 (holotype, K000037101!; isotype, K000037102!).

**Handwriting annotation from the holotype specimens** (Fig. 1B): “Begonia ass. B. Balansaeanae Gagnep. almost aff.?. Flowers white. Leaves very dark green above, glossy, with a metallic lustre, purple nervation, margin crenated. On shady banks and rocks in the jungle. Whole plant glabrous. The contrast between the white flowers and the dark shining leaves amongst which they nestle is very striking”.

#### Revised description.

Plants terrestrial, perennial; stems rhizomatous, 3–10 cm long, 0.2–0.4 cm diam., with fibrous roots on node and 0.5–0.8 cm long internode. Stipules caducous, lanceolate, 1.1–1.3 × 0.4–0.5 cm, margin entire, apex acuminate. Leaf alternate, pliciform, rotund, 10–16 cm diam., margin entire, symmetric on base, usually 4–7-palmatifid venation; adaxially glabrous and serpentinous, green lines and 3–5 spots along each main nerve, abaxially red and pubescent along the main nerves. Petiole 10–18 cm long, densely pubescent. Inflorescences axillary, cymose, dichasial, with separate male and female individuals; peduncles 3–5 cm long; bracts greenish, ovate, 8–9 × 3–4 mm, persistent during flowering. Bracteoles similar to and slightly smaller than bracts; petals white, glabrous on both sides. Male flower: petals 4; outer 2, ovate, 1.2–1.4 × 0.6–0.7 cm, inner 2, elliptic, 0.5–0.6 × 0.2–0.3 cm; androecium actinomorphic, 0.5–0.6 cm diam., filaments free below, anthers oblong, almost equal to the filaments, dehiscent with laterally and obliquely longitudinal slits, connective slightly extended and truncate on the top. Female flower: petals 2, broadly elliptic, 0.9–1× 1.1–1.3 cm; ovary wingless, obtusely 3-hooked, 3-locular, placentation axial, placenta segments thick, 2 per locule, ovules present on both sides of placental branches; styles 3, forked twice, caducous in fruit, stigmas spiralled into a band. Fruit triangular berry-like, pendulous, with an in distinct beak. Flowering Oct. to Nov., Fruiting from the first of Nov. to Oct. of the next year.

#### Distribution.

Only seen in Kachin State, Myanmar.

#### Additional examined specimens.

Upper Burma (=Myanmar): Kachin Hills, 30 November, 1912, collect. Capt. & M. Joppin 4378 (K!); Myanmar, Kachin, Putao, on shaded banks and rocks, 27°20'N, 97°30'E, alt. 900 m, Dec. 10 1937, Kingdon-Ward 13569 (BM!); Myanmar, Kachin State, Putao, Wasadam village, alt. 860 m, 27°30'09"N, 97°11'45"E, near the stream in the Musa forests, occasional, Oct. 15, 2014, Putao Exped. 311 (KUN!, PE!); Myanmar, Kachin State, Putao, Wasadam village, alt. 900 m, 27°30'06"N, 97°11'44"E, along the moist slope in the *Musa* forests, occasional, Oct. 25, 2014, Putao Exped. 1230 (PE!).

#### Discussion.

In Begonia sect. Sphenanthera, *Begonia kingdon-wardii* is unique in the pliciform leaf and female flower with two tepals (Doorenbos et al. 1999; Shui et al. 2002). It is obviously different from *Begonia burkillii* Dunn in B. sect. Sphenanthera and *B. rockii* Irmsch. in B. sect. Platycentrum in the locules of ovary and leaf shape. Morphologically, this species is also similar to *B. gulinqingensis* S. H. Huang & Y. M. Shui (Begonia sect. Diploclinium) in the leaf shape, placentation and fruit shape, but different in its dioecious plant, pliciform leaf, female flower with two sepals and berry fruit. It is also similar to *B. leprosa* (Begonia sect. Leprosae) in the leaf shape, especially the texture of the leaf blade and *B. zhengyiana* Y. M. Shui (Begonia sect. Coelocentrum) in shape of the leaf blade and fruit.

**Figure 2.**
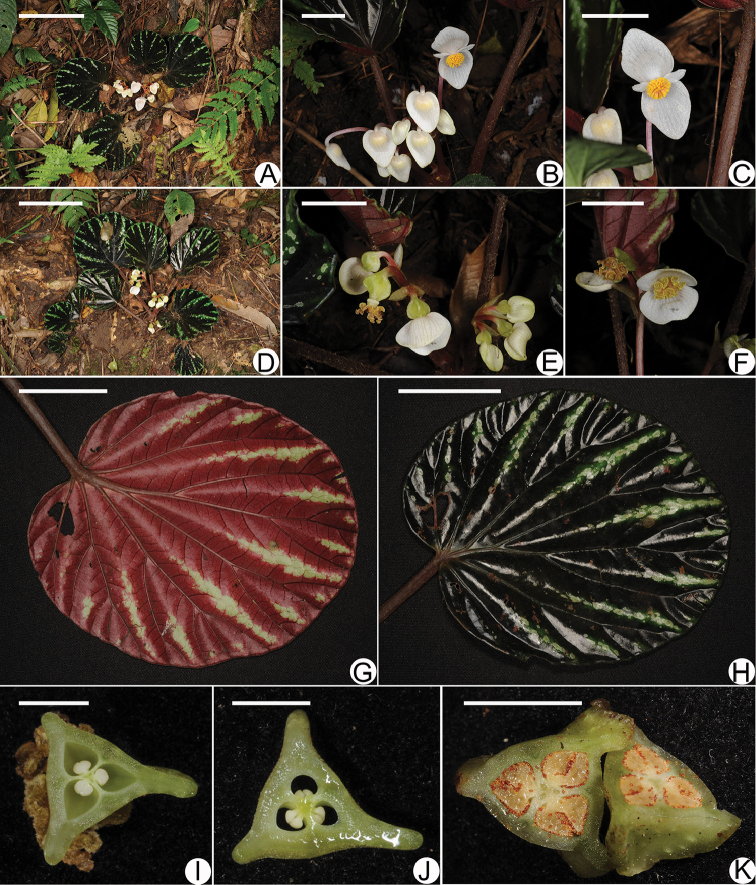
The images of *Begonia kingdon-wardii* Tebbitt (Putao Exped. 311 in PE and KUN) **A** Male plant **B** Male inflorescences **C** Face view of male flower **D** Female plant **E** Female inflorescences **F** Face view of female flower **G** Leaf blade adaxially **H** Leaf blade abaxially **I** Middle section of ovary in flower showing two placenta segments per locule **J** Inferior section of ovary in flower **K** Middle section of mature berry-like fruit showing thick placenta segments. Scale bars: **A, D** 10 cm **B, E** 1 cm **C, F** 1 cm **G, H** 4 cm **I, J, K** 1 cm. All photographed by Yu-Min Shui.

The rediscovery of its living plants provides researchers an opportunity to explore its taxonomic description and horticultural value in North Myanmar. This species with very rare individuals is distributed in a restricted area in Northern Myanmar and grows in the very shady and dark places under the forests. Its flowers are near the ground under the leaves, so that this habit influences the pollination and fruit setting. Another important and interesting habit may be that the fruits need over one year to become mature as some species [*B. handelii* Irmsch. and *B. silletensis* (A. DC.) C. B. Clarke] in Begonia sect. Sphenanthera. Besides, the pliciform leaf of the living plant is difficult to be observed on the holotype (Figs 1, 2). Now, the rediscovery not only reveals the need to undertake more surveys in North Myanmar, but also fills the gap about the deficient data of the species indicated by Tebbitt (2007) and so can bring an amazing plant to mankind for research and horticultural use (Fig. 2).

## Supplementary Material

XML Treatment for Begonia kingdon-wardii
